# Association between the Inflammatory Potential of Diet and Stress among Female College Students

**DOI:** 10.3390/nu12082389

**Published:** 2020-08-10

**Authors:** Leenah Alfreeh, Mahmoud M. A. Abulmeaty, Manal Abudawood, Feda Aljaser, Nitin Shivappa, James R. Hebert, May Almuammar, Yazeed Al-Sheikh, Ghadeer S. Aljuraiban

**Affiliations:** 1 Department of Community Health Sciences, College of Applied Medical Sciences, King Saud University, Riyadh 11362, Saudi Arabia; lalfreeh@ksu.edu.sa (L.A.); mabulmeaty@ksu.edu.sa (M.M.A.A.); malmuammar@ksu.edu.sa (M.A.); 2 Medical Physiology Department, Faculty of Medicine, Zagazig University, Zagazig 44519, Egypt; 3 Department of Clinical Laboratory Sciences, College of Applied Medical Sciences, King Saud University, Riyadh 11362, Saudi Arabia; mabudawood@ksu.edu.sa (M.A.); faljaser@ksu.edu.sa (F.A.); yalsheikh@ksu.edu.sa (Y.A.-S.); 4 Chair of Medical and Molecular Genetics Research, Department of Clinical Laboratory Sciences, College of Applied Medical Sciences, King Saud University, Riyadh 11433, Saudi Arabia; 5 Department of Epidemiology & Biostatistics, University of South Carolina, Columbia, SC 29208, USA; shivappa@email.sc.edi (N.S.); jhebert@sc.edu (J.R.H.); 6 Cancer Prevention and Control Program, University of South Carolina, Columbia, SC 29208, USA

**Keywords:** stress, inflammation, dietary inflammatory index, college students, hs-CRP, PSS

## Abstract

A pro-inflammatory diet may have an adverse influence on stress and inflammatory biomarker levels among college students. The dietary inflammatory index (DII^®^) is a tool used to assess the inflammatory potential of a diet. However, evidence for the association between DII and stress is limited. We examined the association between energy-adjusted DII (E-DII^TM^), high sensitivity-C-reactive protein [hs-CRP], and stress among female college students. This cross-sectional study included 401 randomly selected female students, aged 19–35 years. Data collection included blood, anthropometric measurements, a healthy-history questionnaire, the perceived stress scale (PSS-10), the Saudi food frequency questionnaire (FFQ), and E-DII. Multiple linear regression analyses were used to examine the association between FFQ-derived E-DII score, hs-CRP, and PSS. A higher E-DII score per 1SD (1.8) was associated with a 2.4-times higher PSS score (95% CI: 1.8, 3.1). Higher hs-CRP per 1SD (3.3 mg/L) was associated with a 0.9 (95% CI: 0.7–1.1) times higher PSS score, independent of lifestyle and dietary factors. Our findings indicate that pro-inflammatory diets were highly prevalent among Saudi college students and were associated with higher stress levels. Consideration of the role of stress and focusing on anti-inflammatory foods may be key for healthier dietary habits.

## 1. Introduction

Mental health issues such as stress, anxiety, and depression are an area of concern worldwide [1,2]. The American Psychological Association, the largest scientific organization representing psychology in the United States (US), reported that 78% of adults had increased or unchanged stress levels in the previous five years [2]. Overwhelming stress for long periods of time has been considered a risk factor for depression and other chronic conditions such as cardiovascular diseases (CVDs) and cancer [3,4].

Observational studies conducted in Saudi Arabia revealed that college students, particularly females, had high stress levels (45–65%) [5,6,7]. College students are constrained by long study hours, sleep deprivation, and inadequate personal time, which may lead to stress symptoms that result in adverse consequences on well-being [6]. Previous cross-sectional studies reported that female Saudi students were more vulnerable to high stress levels than their male peers [6,8]. This may be attributed to some conservative social norms of the Saudi community, which can play a role in increasing stress levels among females [9]. Chronic stress can affect food intake and food choices made by college students, making them highly vulnerable to unhealthy dietary habits [10,11]. A previous study reported that while female students generally had healthier dietary habits than their male counterparts, females were more susceptible to making unhealthy food choices (e.g., sweets) as a coping mechanism when experiencing stress [9]. Academic-related stress due to anticipated failure during one’s academic career may increase systemic cortisol levels, which might alter cytokine levels, resulting in immune dysregulation rather than overall immunosuppression [12]. Moreover, food choices can be influenced by stress, which, therefore, may cause unhealthy dietary habits that promote inflammation [13].

Recently, there has been an increased interest in the role of diet as a modulator of the inflammatory process [14,15]. Different individual dietary patterns can exert anti- or pro-inflammatory effects on an individual’s health [14,15]. A recent systematic review and meta-analysis of longitudinal and cross-sectional studies assessed the association between the inflammatory potential of diet and the risk of mental health disorders [14]. A significant association was observed between a pro-inflammatory dietary pattern, such as the Western diet (energy-dense, rich in saturated fat, sugar, processed foods, etc.), and an increased risk of diagnosis or symptoms of mental health disorders compared to those with an anti-inflammatory dietary pattern, such as the Mediterranean diet (rich in olive oil, fruits, vegetables, etc.) [14].

The dietary inflammatory index (DII^®^) is a validated tool used to assess the inflammatory potential of an individual’s diet based on the overall inflammatory properties of food components, such as macronutrients, micronutrients, flavonoids, and several other bioactive compounds [16]. The development of the DII has been published in detail elsewhere [16]. Briefly, it is based on an extensive search of cellular, animal, and observational literature assessing the effect of diet on inflammation [16]. Previous investigations conducted among college students and shift workers (e.g., police officers) that used the DII to explore the association between the inflammatory potential of diet and stress yielded contradictory results [17,18] due to several limitations, including the use of less focused stress scales [17], the absence of objective measures of inflammatory biomarkers [17], and different dietary assessment methods.

Whether the inflammatory potential of diet is associated with stress in particular remains unclear, and further research is warranted. We hypothesized that a positive relationship exists between the inflammatory potential of diet (as measured by E-DII) and stress. The aim of the present study was to investigate the E-DII and objective measures of inflammatory biomarkers (high sensitivity-C reactive protein (hs-CRP)) in association with stress (as measured by perceived stress scale version 10 [PSS-10]) in a sample of Saudi female students of King Saud University (KSU).

## 2. Materials and Methods

### 2.1. Study Design and Setting

The present cross-sectional investigation was conducted at the female campus of KSU, Riyadh, Saudi Arabia between February and May of 2019. The study protocol was approved by the Institutional Review Board of the Deanship of Scientific Research (ref. no. 19/0105/IRB). Prior to the initiation of this study, a pilot study was conducted among 30 other participants to ensure the accurate progression of the study procedures, such as assessment procedures, allocated time for each participant, etc. Data for these participants were left unreported in the final study samples as they were used for standardization purposes.

### 2.2. Study Sample

The study sample included 401 randomly selected medical and non-medical students, aged 19–35 years. Students were invited to participate in our study through advertisements, social media networks, and word-of-mouth. We excluded individuals who had been diagnosed with chronic diseases (e.g., CVD and diabetes mellitus), inflammatory diseases (such as rheumatoid arthritis), or infections [19]; those who were on antidepressant medications [20]; taking high doses (>300 mg/day) of nonsteroidal anti-inflammatory medications (such as aspirin) [21]; were on hormonal contraceptives (such as birth control pills) [22]; or were pregnant [23].

The data were collected during the 2018/2019 academic year over a 4-month period. Data regarding demographic characteristics, dietary habits, and anthropometric measures were collected at the clinic of the Community Health Sciences Department of the College of Applied Medical Sciences, KSU. Afterward, blood samples for biochemical analysis were obtained in the phlebotomy room of the Laboratory Department of the same college. Each participant completed the visit in approximately one hour. Written informed consent was obtained from all participants. The participants had the right to withdraw from the study at any time.

### 2.3. Sample Size

Given a total population of 16,818 female students at KSU with stress levels ranging between 45% and 65% [5,6,7], a sample size of 400 students would be needed for sufficient statistical power, maintaining a margin of missing data set at 5% with a confidence level of at least 95%.

### 2.4. Study Tools

#### 2.4.1. Health History Questionnaire

An interview-based questionnaire was used to collect data on general sociodemographic characteristics (age, nationality (Saudi, non-Saudi)), marital status (single, married), education level (bachelor, master, PhD), academic level (level 1–3, level 4–6, level 7–9), college (medical or non-medical), family income per month categorized according to the Saudi General Authority of Statistics [24], medical history (presence of chronic medical conditions (yes, no)), lifestyle and dietary habits (sleeping hours/day (less than 6 h, 6–7 h, >7 to ≤10 h, more than 10 h)) as per the National Sleep Foundation [25], frequency of physical activity per week (daily, 4–6 times, less than 4 times)), duration of physical activity (minutes per day), and previous weight reduction diet (yes, no).

#### 2.4.2. Dietary Assessment

During the clinic visit, trained interviewers used a standardized protocol to query participants on their food and beverage consumption over the preceding 12 months using a validated Saudi food frequency questionnaire (FFQ) [26]. The FFQ consists of 10 sections with 133 food items. Food groups were defined based on previously published data [27] including whole grains, fruits, vegetables, nuts, legumes, vegetable oils, tea/coffee, fruit juices, sugar-sweetened beverages, refined grains, potatoes, sweets and desserts, dairy products, eggs, fish or shellfish, total meat, and miscellaneous animal-based foods, e.g., hamburger, pizza. Furthermore, local foods have been added to each food category according to their type. The FFQ also includes questions regarding fast food consumption, as well as artificial sweeteners, different types of fats, and salt added by the participants. Additional data regarding nutrients and spices (such as garlic, ginger, pepper, thyme/oregano, rosemary, turmeric, and saffron) required for the assessment of the DII were included. Information on food consumption and the portion size estimation for each food item was individually obtained by interviewers using household measures, food modules, and pictures. The nutritional content of the foods was assessed using the US Department of Agriculture food composition tables [28]. Dietary data were entered into NutriBase version 20 (CyberSoft Inc., Phoenix, AZ, USA); for local foods, the 1st version of the Arabic program; National King Fahad Library 5716/1427(-Riyadh, Saudi Arabia, 2007) was employed, which has been used in a previous study [29].

#### 2.4.3. Dietary Inflammatory Index

The development of the DII has been described in detail elsewhere [16]. The inflammatory potential of the diet was calculated by computing the amounts of nutrients provided by the FFQs. The mean intake of each food item was translated into statistical indices (*Z*-scores and centered percentiles), which were multiplied by their respective coefficients (overall food parameter-specific inflammatory effect scores) to calculate the DII score for each food parameter. Scores were summed to obtain the overall DII score for each participant. A positive DII score corresponds to a pro-inflammatory dietary habit and a negative DII score translates to an anti-inflammatory dietary habit. The following 36 food parameters were available to calculate the DII in the present study: carbohydrates, fat, protein, cholesterol, trans fat, saturated fatty acids, monounsaturated fatty acids, polyunsaturated fatty acids, n-3 fatty acids, n-6 fatty acids, vitamin B12, vitamin B6, vitamin A, vitamin C, vitamin D, vitamin E, riboflavin, thiamine, iron, folic acid, zinc, magnesium, selenium, niacin, fiber, caffeine, green/black tea, garlic, onion, ginger, rosemary, pepper, thyme/oregano, turmeric, and saffron.

#### 2.4.4. hs-CRP Test

A total of 2 mL of blood sample was obtained from each participant by a trained nurse during the daytime, usually between 8:00 a.m. and 3:00 p.m. All participants underwent a relaxation period for approximately 15 min before blood samples were drawn. Serum was separated by centrifugation for 10 min within 1 h of collection and was kept frozen at −80 °C for blood biochemical analysis later at the end of sample collections. hs-CRP levels were measured via enzyme-linked immunosorbent assay (ELISA) using human High Sensitivity Human C-Reactive Protein (hs-CRP) ELISA kit from (Aviva Systems Biology, OKBA00016, San Diego, CA, USA) according to the manufacturer’s protocol. The hs-CRP assays were calibrated against the international standards of the Centers for Disease Control and Prevention (CDC) using the kit manufacturer’s protocol. All samples and standards were run in duplicate to ensure accurate results. The levels of hs-CRP were assessed in 289 randomly selected participants to minimize cost. According to the CDC classification for identifying individuals with a higher risk of CVD, an hs-CRP level >3 mg/L is considered high, 1–3 mg/L is considered moderate, and <1 mg/L is considered low [30]. Biochemical analysis was performed in collaboration with the central laboratory of KSU.

#### 2.4.5. Perceived Stress Scale

Stress levels were assessed using the PSS-10 (Arabic version), a validated stress measurement tool with adequate psychometric properties regarding reliability and validity among different populations [31]. The scale consists of 10 items that measure perceived stressful experiences over the preceding month on a 5-point Likert scale ranging from never (0), almost never (1), sometimes (2), fairly often (3), and very often (4). The total PSS score is calculated by reversing the scores for questions (4, 5, 7, and 8) to (0 = 4, 1 = 3, 2 = 2, 3 = 1, 4 = 0) and summing the values. We divided individual PSS scores into tertiles: values of 4–17 reflect low stress levels, values of 18–23 reflect moderate stress levels, and values of 24–36 reflect high levels of perceived stress. The estimated time to complete the PSS-10 questionnaire was approximately 8 min per participant.

#### 2.4.6. Anthropometric Measures

Trained staff measured each participant’s weight (to the nearest 0.1 kg) and height (to the nearest 0.1 cm) twice, using the same mechanical weight beam physician scale and stadiometer (Detecto, Webb City, MO, USA) in the standing position. These were averaged to calculate the body mass index (BMI) (in kg/m^2^) by dividing the weight by the square of the height. Individuals with a BMI of (25–29.9 kg/m^2^) were classified as overweight, and those with a BMI (≥30 kg/m^2^) were classified as obese according to the World Health Organization (WHO) guidelines [32]. Waist circumference was measured to the nearest 0.1 cm using a non-stretchable tape measure at the mid-point between the lowest rib and the iliac crest, and hip circumference was measured around the widest portion of the buttocks as per the WHO guidelines. Measurements were done twice and were averaged to calculate the waist-to-hip ratio by dividing the waist over the hip circumference. A waist-to-hip ratio of >0.85 is considered as the cut-off value to identify abdominal obesity among females, as per the WHO guidelines [33]. Bioelectrical impedance analysis (InBody 770; InBody Inc., Cerritos, CA, USA) was used to obtain data on body composition according to the manufacturer’s protocol.

### 2.5. Statistical Analysis

Descriptive statistics are presented as the mean and standard deviation (SD) for continuous variables and as frequencies and percentages for binary and categorical variables.

The baseline characteristics of participants are presented as energy-adjusted DII (E-DII) tertiles (≤3.75, 3.76–4.40, and >4.40). A linear age-adjusted model was used to assess the linearity of the investigated relations.

Pearson’s partial correlation analyses with adjustment for age was used to assess the correlations among E-DII, anthropometric characteristics, lifestyle variables, and outcome variables.

Multivariable linear regression analyses were performed to assess the association between the E-DII, hs-CRP, and PSS per 1SD (E-DII = 1.8, hs-CRP = 3.3 mg/l). The models were adjusted for lifestyle and dietary factors that could confound the association. Model 1 was a simple model adjusted for age [34]. Model 2 (the main model) was adjusted for variables in model 1 and marital status [34], education level [34], college (medical or non-medical) [34], family income [35], presence of chronic medical conditions [4], hours spent sleeping per day [36], duration of physical activity [37], and previous weight reduction diet [38]. Model 2a was adjusted for all the variables in model 2 and BMI [38]. Model 2b was adjusted for all the variables in model 2 and waist-to-hip ratio [38]. All statistical analyses were performed using SAS version 9.4 (SAS Institute Inc., Cary, NC, USA). Analysis items with *p*-value < 0.05 were considered statistically significant.

## 3. Results

### 3.1. Participants’ Characteristics

The mean body weight, BMI, and waist-to-hip ratio of participants were 60.29 ± 13.85 kg, 24.05 ± 6.04 kg/m^2^, and 0.75 ± 0.17, respectively (Table S1). Regarding body composition, (79.8%) of study participants had mean fat and muscle percentages of 35.11 ± 7.72% and 53.47 ± 13.59%, respectively. The mean hs-CRP was 2.62 ± 2.31 mg/L, which is considered above the CDC cut-off criteria. The mean E-DII score was 3.90 ± 1.08 (range: −0.09 to 6.1), indicating an overall pro-inflammatory diet. Further, most of our participants (57%) were classified as moderately stressed, and about (30%) were classified as highly stressed with a mean PSS score of 20.12 ± 6.13 (Table S1).

Compared to participants with a low E-DII, those in the high E-DII tertile had a higher BMI, body fat percentage, PSS score, hs-CRP value, and had a significantly lower body muscle percentage (Table 1).

Participants with a high E-DII score had higher intakes of total energy, total fat, total cholesterol, saturated fatty acids, trans fat, and lower intakes of polyunsaturated fatty acids, total carbohydrate, and total fiber compared to those with low E-DII scores (Table S2). Regarding micronutrients, compared to participants with a low E-DII score, those with a high E-DII score had lower intakes of vitamins A, C, E, D, B12, and B6; thiamine; riboflavin; folic acid; niacin; zinc; magnesium; and selenium. The intakes of other food items such as green/black tea, garlic, onion, and thyme/oregano were lower among participants with a high E-DII score compared to those with a low E-DII score (Table S2).

Pearson’s partial correlation coefficient of the relationship between E-DII scores and PSS scores was (*r* = 0.46). E-DII was positively correlated with the hs-CRP level (*r* = 0.46), BMI (*r* = 0.33) and inversely correlated with muscle percentage (*r* = −0.36) (Table S3).

### 3.2. Associations between E-DII, hs-CRP and PSS Score

A higher E-DII score per SD (1.8) was associated with a 2.7 (95% CI: 2.3–3.3) times higher PSS score after adjustment for lifestyle and dietary factors (Model 2; Table 2). hs-CRP higher per SD (3.3 mg/l) was associated with a 0.9 (95% CI: 0.7–1.1) times higher PSS score, independent of lifestyle and dietary factors (Model 2; Table 2). These results were independent of BMI and waist-to-hip ratio.

## 4. Discussion

### 4.1. Main Findings

In this cross-sectional study of female students, we observed a positive association between E-DII score, hs-CRP, and stress, assessed using the PSS score. These associations persisted after adjustment for lifestyle and dietary factors, BMI, and waist-to-hip ratio. We also found that among our participants, the average E-DII scores indicated an overall pro-inflammatory dietary habit. In addition, the average hs-CRP level among our participants was considered high (2.62 ± 3.31 mg/L) according to the CDC cut-off value [30] and was higher among participants in the high E-DII tertile than the low. In addition, the prevalence of participants classified as moderately or severely stressed was unexpectedly high 87%. To the best of our knowledge, the present study is the first to examine the association between the inflammatory potential of diet (assessed via the E-DII) and stress (assessed via the PSS-10) in addition to using objective measures of inflammatory biomarkers in a sample of Saudi females.

### 4.2. Comparison with other Studies

We found a positive association between pro-inflammatory dietary patterns and stress levels. A previous systematic review and meta-analysis demonstrated a significant association between the pro-inflammatory dietary pattern and an increased risk of diagnosis or symptoms of mental health disorders as compared to the anti-inflammatory dietary pattern (*p* < 0.001) [14]. Our findings are comparable to those reported by Shivappa et al., observing a significant, positive association between DII score and depression and anxiety stress scores among female college students [17]. Females with the most pro-inflammatory diets had higher stress levels than those with the most anti-inflammatory diets [17]. In line with these findings, a significant association was found between a pro-inflammatory diet (reflected by a higher DII score) and a higher risk of depression in a cohort study of 15,093 Spanish university graduates with a follow-up of approximately 9 years (*p* = 0.01) [39]. Participants with the highest DII scores were at a 150% higher risk of depression compared to those with the lowest DII scores [39]. In contrast to the previous findings, some studies found no association between DII scores and stress or depression [18,40]. In a longitudinal cohort study including 3535 French adults with a follow-up of 13 years, no significant association was observed between DII score and the occurrence of depressive symptoms among females (*p* = 0.6) [40]. This may be due to methodological limitations, including different study designs, the use of different stress scales, absence of objective measures of inflammatory biomarkers, and varied dietary assessment methods.

The mean E-DII score obtained in our study was 3.90 ± 1.08 based on 37 food parameters. These values, specifically in the 90th percentile, reflect a strong pro-inflammatory dietary pattern among our participants. Previous investigations reported different ranges of DII, derived from a variety of food components used in the original DII scoring system. A study including 110 college students in the US also reported high DII scores, with the upper bound of the lowest tertile = 2.8 to 4.6 based on 30 food parameters [41]. In a population of 3363 Middle Eastern adults, the DII ranged between −5.55 and +4.61 based on 27 food parameters [42]. In a Spanish cohort of 15,093 university graduates, the DII ranged from −3.16 to +0.66 based on 28 food parameters [39]. However, our results (range: −0.09 to 6.1) were higher and reflect an overall pro-inflammatory dietary habit compared to other population samples. Additionally, body composition analysis revealed that most participants in our study had a high fat percentage according to the American Council on Exercise [43]. Furthermore, their average BMI was (24.05 ± 6.04 kg/m^2^); classifying their average as being close to overweight [32]. These findings indicate that most participants in our study have poor dietary habits. Results of a recent cross-sectional study conducted among Saudi college students found that students were exposed to unhealthy fast-food restaurants on the college campus [44]. About 32% of students reported that during the examination period, they did not have sufficient time to cook healthy foods, while a majority of them (67%) reported that their food choices would be affected when they were under stress, leading them to choose more convenient foods such as fast foods, sweets, and soda [44], which are classified as pro-inflammatory foods. Several studies conducted worldwide have also observed unhealthy dietary habits among college students [10,11].

Our results also revealed a positive association between hs-CRP and stress levels, independent of lifestyle and dietary factors, BMI, and waist-to-hip ratio. Additionally, compared to participants in the lowest E-DII tertile, those in the high E-DII tertile had higher hs-CRP. Unhealthy dietary habits (such as the Western diet) have been positively associated with elevated hs-CRP levels; individuals with the most pro-inflammatory dietary habits are 173% more likely to have elevated hs-CRP levels (>2 mg/L) compared with individuals with the most anti-inflammatory diets [15,45]. In contrast, following healthy dietary habits (such as the Mediterranean diet) for 12 months led to a reduction in CRP level by 0.65 mg/L (*p* = 0.02) [46]. The elevation in the levels of inflammatory biomarkers through pro-inflammatory dietary habits may alter the HPA axis, interact with neurotransmitters (serotonin, dopamine, noradrenaline, and glutamate), and alter neuroplasticity and neuroendocrine function, which in turn affect the immune system and cause low-grade inflammation, contributing to the development of mental health disorders [47]. Furthermore, gut microbiota, which is regulated by dietary patterns, can activate the neural pathway and thus the central nervous system [48].

One of the most important findings is that 57% of our participants were classified as moderately stressed and approximately 30% were classified as highly stressed. We found a weak but positive correlation between the PSS score and waist-to-hip ratio. Additionally, the PSS score was strongly correlated with fat percentage. It is possible that exposure to stress activates individuals’ emotional responses, leading them to increase their energy intake through the consumption of foods that are high in fat and sugar; and this is particularly evident among college students [11,49]. The association between stress, highly palatable foods, and obesity remains unclear [50]. In response to stress, the hypothalamic pituitary adrenal (HPA) axis is stimulated and interferes with the reward system of the brain [50]. Consumption of high-fat, sweet foods causes frequent stimulation of the reward system of the brain [50]. The neurological adaptation to the frequent stimulation then leads to this overeating phenomenon [50]. Furthermore, the HPA axis is activated in response to stressful situations, including academic stress, leading to the release of cortisol (a stress hormone) and leptin (an anti-obesity hormone) [51]. Notably, the activation of the HPA axis due to stress tends to result in higher glucocorticoid levels in females than in males [52]. Moreover, stress, as well as increased consumption of highly palatable foods, has been assumed to increase the amount of abdominal fat through increased secretion of neuropeptide Y (NPY) in the plasma [53]. Under conditions of stress, NPY is released by the sympathetic nervous system, which in turn stimulates the growth of adipocytes and causes an increase in the visceral fat mass [53].

### 4.3. Strengths and Limitations

The present study had several strengths. The sample size was sufficiently representative of female students of KSU. We also used validated assessment tools (e.g., FFQ, PSS-10, and E-DII) administered by trained interviewers, instead of self-administered tools, following a robust and rigorous protocol. Anthropometric measurements were done twice by trained researchers in accordance with the WHO guidelines. The inflammatory biomarker, hs-CRP, was available to support the result of E-DII scores objectively, in contrast to the previous literature. We calculated the E-DII based on 37 food parameters; only 8 food parameters were missing.

The present study had some limitations, such as its cross-sectional design, such that temporality could not contribute to causal inference between E-DII score and stress. Although we considered several confounding factors, residual confounding caused by inaccurate measurements cannot be ruled out. Additionally, misclassification while obtaining dietary data and reporting bias are unavoidable. Because the study participants were young female college students, caution must be exercised in generalizing the findings of the present study, since they might not be representative of the general Saudi population.

## 5. Conclusions

Our findings may have important clinical implications regarding the role of improved dietary habits in stress management. Furthermore, this study may provide guidance regarding nutrition interventions to reduce the inflammatory potential of diet by focusing on anti-inflammatory foods, which in turn reduce the risk of certain chronic diseases. Unhealthy dietary habits constitute an important public health concern that requires control, particularly at a young age. It is necessary to confirm whether adherence to pro-inflammatory dietary habits increases stress levels or whether stressed participants are more likely to choose unhealthier foods in future randomized controlled trials.

## Figures and Tables

**Table 1:** Clinical and demographic characteristics of study participants by E-DII adjusted for age, n = 401 ^1^.

Variable	Tertile 1(−0.09 to 3.75)	Tertile 2(3.76 to 4.40)	Tertile 3(4.41 to 6.1)	*p*-Value
	E-DII			
n	133	134	134	
Age (y)	21.6 (21.0–22.1)	21.3 (20.8–21.9)	22.1 (21.5–22.6)	0.45
*Nationality*				
Saudi	91%	94.8%	93.3%	
*Marital status*				
Single	91.7%	92.5%	91.8%	
*Education level*				
Bachelor	88.7%	89.6%	89.6%	
Master	10.5%	9.7%	9.0%	
PhD	0.8%	0.7%	1.5%	
*Academic level*				
Level 1–3	21.1%	26.1%	29.1%	
Level 4–6	43.6%	50.0%	50.0%	
Level 7–9	53.3%	23.9%	20.9%	
*College*				
Medical	29.3%	47.0%	41.8%	
Non-medical	70.7%	53.0%	58.2%	
*Family income per month (SR)*				
<5000	3.0%	6.7%	4.5%	
5000 – < 10,000	29.3%	21.6%	23.1%	
10,000 – < 19,000	31.6%	29.9%	28.4%	
≥19,000	36.1%	41.8%	44.0%	
Presence of medical conditions ^2^	1.5%	5.2%	14.2%	
Sleeping (h/d)	2.3 (2.2–2.5)	2.3 (2.2–2.5)	2.2 (2.1–2.4)	
*Sleeping (h/d)*				
<6 h	14.3%	17.2%	19.4%	
6–7 h	41.4%	36.6%	41.0%	
> 7 – ≤ 10 h	41.4%	44.0%	35.1%	
>10 h	3.0%	2.2%	4.5%	
Frequency of physical activity (times/w)	4.6 (4.3–4.9)	4.7 (4.4–5.0)	5.0 (4.6–5.3)	
Duration of physical activity (min/d)	55.1 (47.7–62.5)	61.5 (54.1–68.9)	66.7 (59.3–74.0)	
Previous weight reduction diet	24.1%	38.1%	48.5%	
BMI (kg/m^2^)	21.7 (20.7–22.6)	24.4 (23.4–25.4)	26.0 (25.1–27.0)	<0.0001
Waist-to-hip ratio	0.73 (0.71–0.76)	0.76 (0.73–0.79)	0.75 (0.72–0.78)	<0.46
Body fat (%)	31.74 (30.51–32.89)	35.39 (34.16–36.62)	38.17 (36.94–39.41)	<0.0001
Body muscle (%)	59.69 (57.53–61.85)	52.98 (50.82–55.14)	47.80 (45.63–49.96)	<0.0001
PSS score	16.6 (15.6–17.5)	21.3 (20.3–22.2)	22.5 (21.6–23.5)	<0.0001
Hs-CRP (mg/L) ^3^	1.23 (0.69–1.78)	2.26 (1.66–2.85)	4.77 (4.15–5.39)	<0.0001

^1^ Values are mean (95% CI) or %. Energy adjusted dietary inflammatory index (E-DII), Saudi riyals (SR), Body mass index (BMI), Perceived stress scale (PSS), High sensitivity C reactive protein (hs-CRP). ^2^ Medical condition includes chronic constipation, gastroesophageal reflux disease, prolactinoma, vitamin D deficiency, polycystic ovary syndrome, and hypothyroidism.^3^ Hs-CRP was collected from 289 participants.

**Table 2:** Estimated mean differences in PSS score associated with 1SD higher E-DII score and hs-CRP in KSU female participants, n = 401 ^1,2^.

Variable	PSS Score		
	Difference in PSS	(95% CI)	*p*-Value
E-DII score			
Model 1	3.1	(2.7, 3.6)	<0.001
Model 2	2.4	(1.9, 3.1)	0.01
Model 2a	2.2	(1.6, 2.8)	0.03
Model 2b	2.3	(1.5, 2.7)	0.03
hs-CRP ^3^			
Model 1	0.9	(0.7, 1.1)	<0.0001
Model 2	0.9	(0.7, 1.1)	<0.0001
Model 2a	0.8	(0.7, 1.0)	<0.0001
Model 2b	0.9	(0.7, 1.1)	<0.0001

^1^ Energy-adjusted dietary inflammatory index (E-DII), perceived stress scale (PSS), high sensitivity C-reactive protein (hs-CRP). Values are presented as mean (95% CI). ^2^ Model 1 is a simple model adjusted for age; model 2 is model 1 additionally adjusted for marital status, education level, college, family income, presence of chronic medical conditions, hours spent sleeping per day, duration of physical activity, and previous weight reduction diet; model 2a is model 2 additionally adjusted for BMI; model 2b is model 2 additionally adjusted for waist-to-hip ratio. 1 SD = 1.8 for E-DII and 3.3 mg/L for hs-CRP. ^3^ Hs-CRP was collected from 289 participants.

## Supplementary Materials

The following are available online at https://www.mdpi.com/2072-6643/12/8/2389/s1, Table S1: Descriptive characteristics of study participants (anthropometric measurements, BP, biochemical data, PSS score, and E-DII score), Table S2: Dietary intakes of study participants across tertiles of E-DII, Table S3: Pearson partial correlation between E-DII, anthropometrics, lifestyle variables and outcome variables for our study participants.
