# Supplementary material for: Association between the Inflammatory Potential of Diet and Stress among Female College Students
Source: Nutrients. 2020 Aug 10;12(8):2389. doi: 10.3390/nu12082389 (PMC7468951; doi:10.3390/nu12082389)
Supplement: Supplementary file 1 [file nutrients-12-02389-s001.pdf]

Table S1. Descriptive characteristics of study participants (anthropometric measurements, biochemical data, PSS score, and E-DII score), n=401 [1]

Characteristics	Mean	± SD
Weight (kg)	60.29	±13.85
Height (cm)	158.62	±5.59
BMI (kg/m²)	24.05	±6.04
Waist (cm)	75.05	±11.49
Hip (cm)	101.49	±12.70
Waist-to-hip ratio	0.75	±0.17
Fat (%)	35.11	±7.72
Muscle (%)	53.47	±13.59
Hs-CRP (mg/l) [2]	2.62	± 3.31
PSS score	20.12	± 6.13
E-DII	3.90	± 1.08

[1]Data are mean ± standard deviation (SD)

Body mass index (BMI), High sensitivity C reactive protein (hs-CRP), Perceived stress scale (PSS), Energy adjusted dietary inflammatory index (E-DII)

[2]Hs-CRP were collected from 289 participants

Table S2. Dietary intakes of study participants across tertiles of E-DII, n=401 [1,2]

Variable	Tertile 1 (≤ 3.75)	Tertile 2 (3.76-4.40)	Tertile 3 (>4.40)	P-value
	E-DII			
n	133	134	134	
Energy (Kcal/day)	1710 (1607 – 1814)	2047 (1944 – 2151)	2393 (2289 – 2496)	< 0.0001
Carbohydrate (% E) [2]	58.1 (54.2 – 62.0)	49.3 (45.4 – 53.3)	45.3 (41.4 – 49.2)	< 0.0001
Protein (% E) [2]	21.9 (20.0 – 23.8)	21.8 (19.9 – 23.7)	21.9 (20.0 – 23.8)	0.98
Fat (% E) [2]	20.9 (18.8 – 22.0)	19.6 (17.5 – 21.6)	23.9 (21.8 – 29.6)	0.01
SFA (% of total fat)	13.0 (12.5 – 15.4)	11.6 (10.1 – 13.1)	13.8 (12.3 – 15.2)	0.05
PUFA (% of total fat)	4.6 (3.8 – 5.3)	3.3 (2.6 – 4.0)	2.6 (1.9 – 3.3)	0.01
MUFA (% of total fat)	3.9 (3.5 – 4.3)	3.4 (3.0 – 3.9)	3.3 (2.9 – 3.7)	0.13
<u>Nutrients (per 1000 kcal)</u>				
Cholesterol (mg)	96.3 (88.7 – 104.0)	105.4 (97.8 – 113.0)	120.5 (112.9 – 128.2)	< 0.0001
Trans fat (mg)	1.2 (1.1-1.3)	1.3 (1.2-1.3)	1.5 (1.4-1.6)	< 0.0001
n-3 Fatty acids (gm)	0.11 (0.1 – 0.1)	0.11 (0.1 – 0.1)	0.11 (0.1 – 0.1)	0.69
n-6 Fatty acids (gm)	0.8 (0.7 – 0.9)	0.9 (0.9 – 1.1)	0.9 (0.8 – 0.9)	0.12
Fiber (gm)	8.1 (7.7 – 8.5)	6.1 (5.7 – 6.5)	5.2 (4.8 – 5.6)	< 0.0001
Vit A (RE)	107.9 (95.8 – 120.0)	83.5 (71.4 – 95.5)	74.9 (62.8 – 87.0)	0.0005
Vit C (mg)	10.4 (9.6 – 11.2)	9.6 (8.8 – 10.4)	7.9 (7.1 – 8.7)	< 0.0001
Vit E (mg)	2.1 (1.8 – 2.3)	1.5 (1.3 – 1.7)	1.4 (1.2 – 1.6)	< 0.0001
Vit D (μg)	2.0 (1.8 – 2.2)	1.3 (1.1 – 1.5)	1.3 (1.1 – 1.6)	< 0.0001
B12 (μg)	0.1 (0.1 – 0.2)	0.1 (0.1 – 0.1)	0.1 (0.1 – 0.1)	0.01
B6 (mg)	0.1 (0.1 – 0.1)	0.1 (0.1 – 0.1)	0.1 (0.1 – 0.1)	< 0.0001
Thiamine (mg)	0.1 (0.1 – 0.1)	0.1 (0.1 – 0.1)	0.1 (0.1 – 0.1)	< 0.0001
Riboflavin (mg)	0.1 (0.1 – 0.1)	0.1 (0.1 – 0.1)	0.1 (0.1 – 0.1)	< 0.0001
Folic acid (μg)	117.4 (104.9 – 130.0)	91.2 (78.7 – 103.7)	80.1 (67.6 – 92.7)	0.0002
Niacin (mg)	1.2 (1.1 – 1.4)	0.9 (0.8 – 1.1)	0.8 (0.6 – 0.9)	< 0.0001
Iron (mg)	1.4 (1.3 – 1.6)	1.5 (1.3 – 1.6)	1.3 (1.2 – 1.5)	0.37
Zinc (mg)	1.5 (1.3 – 1.6)	1.2 (1.1 – 1.4)	1.1 (1.0 – 1.2)	0.0008
Mg (mg)	54.8 (49.7 – 59.9)	46.7 (41.7 – 51.8)	42.8 (37.8 – 47.9)	0.01
Selenium (μg)	11.1 (10.3 – 11.9)	8.6 (7.8 – 9.4)	7.7 (6.9 – 8.5)	< 0.0001
Caffeine (gm)	10.0 (6.9 – 13.1)	9.7 (6.6 – 12.8)	6.2 (3.1 – 9.3)	0.17
Green/black tea (gm)	137.3 (118.2– 156.3)	131.8 (112.7– 150.7)	95.5 (76.5 – 114.5)	0.005
Garlic (gm)	2.3 (2.0 – 2.6)	1.9 (1.6 – 2.2)	1.2 (0.9 – 1.5)	< 0.0001
Onion (gm)	24.7 (21.2 – 28.2)	21.8 (18.3 – 25.3)	15.3 (11.8 – 18.8)	0.0008
Ginger (gm)	3.2 (0.9 – 5.5)	2.6 (0.4 – 4.9)	2.9 (0.6 – 5.2)	0.94
Rosemary (mg)	0.01 (-0.04 – 0.06)	0.06 (0.02 – 0.11)	0.04 (-0.01 – 0.09)	0.30
Thyme/oregano (mg)	3.5 (2.6 – 4.4)	2.6 (1.7 – 3.5)	1.5 (0.6 – 2.4)	0.01
Pepper (gm)	2.4 (1.8 – 3.1)	2.4 (1.7 – 3.0)	1.9 (1.3 – 2.6)	0.53
Saffron (gm)	0.9 (0.7 – 1.1)	0.8 (0.6 – 1.0)	0.7 (0.5 – 0.9)	0.46
Turmeric (mg)	0.3 (0.1 – 0.4)	0.3 (0.1 – 0.5)	0.5 (0.3 – 0.7)	0.11
<u>Food Groups (gm/1000 kcal)</u>				
Whole grains	34.6 (25.2 – 44.0)	41.1 (31.7 – 50.5)	35.2 (25.8 – 44.6)	0.57
Fruits	142.8 (111.6 – 174.1)	156.3 (125.1 – 187.5)	123.2 (92.0 – 154.4)	0.33
Vegetables	278.5.9 (232.6 – 324.4)	251.1 (205.3 – 296.9)	221.3 (175.5 – 267.1)	0.22
Nuts	21.1 (15.5 – 26.8)	12.4 (6.8 – 18.0)	12.5 (6.8 – 18.1)	0.05
Legumes	27.4 (21.7 – 33.1)	27.2 (21.4 – 32.9)	19.3 (13.6 – 25.0)	0.08
Vegetables oil	22.2 (15.2 – 29.2)	29.0 (22.0 – 35.9)	36.9 (30.0 – 43.9)	0.01
Tea and coffee	345.1 (284.4 – 405.8)	257.3 (196.7 – 317.9)	225.0 (164.3 – 285.6)	0.02

Fruit juices	48.8 (33.8 – 63.7)	43.9 (29.0 – 58.9)	52.4 (37.4 – 67.4)	0.73
Refined grains	227.8 (201.1 – 254.6)	181.7 (155.0 – 208.4)	146.2 (119.4 – 172.9)	0.0001
Potatoes	42.3 (33.6 – 51.0)	41.9 (33.2 – 50.6)	36.7 (28.0 – 45.4)	0.60
Sugar sweetened beverages	51.3 (35.2 – 67.4)	39.4 (23.3 – 55.5)	43.8 (27.7 – 59.9)	0.58
Sweets and desserts	40.7 (34.7 – 46.6)	34.4 (28.5 – 40.5)	34.5 (28.5 – 40.5)	0.25
Dairy products	199.0 (171.2 – 226.9)	159.0 (131.2 – 186.8)	152.8 (125.0 – 180.7)	0.04
Egg	24.3 (19.8 – 28.7)	18.2 (13.8 – 22.6)	15.3 (10.8 – 19.7)	0.02
Fish and seafood	16.0 (12.6 – 19.4)	16.1 (12.7 – 19.5)	11.2 (7.8 – 14.6)	0.07
Meat	40.0 (47.8 – 62.3)	47.2 (40.0 – 54.4)	56.0 (37.7 – 52.2)	0.05
Miscellaneous animal-based foods	39.4 (4.3– 59.0)	45.3 (4.3 – 53.7)	50.6 (4.3 – 47.7)	0.17

[1]Values are mean (95% CI)

[2] Percentage of daily energy intake (% E)

Saturated fatty acid (SFA %), Polyunsaturated fatty acid (PUFA %), Monounsaturated fatty acid (MUFA %), magnesium (Mg)

Table S3. Pearson partial correlation between E-DII, anthropometrics, lifestyle variables and outcome variables for our study participants, n = 401[1]

Variable	PSS score	E-DII	BMI (kg/m2)	Waist-to-hip ratio	Fat %	Muscle %
Family income (SR)	-0.06	-0.01	0.01	0.02	0.00	-0.03
Previous weight loss diet	-0.12	-0.23	-0.43	-0.12	-0.44	0.22
Special weight loss diet	0.07	0.18	0.38	0.17	0.37	-0.21
Previous weight loss / kg	0.06	0.11	0.33	0.07	0.29	-0.09
Duration of PA (min/day)	0.06	0.09	-0.02	-0.07	0.02	-0.05
The intensity of PA	-0.01	0.04	-0.08	-0.08	-0.05	0.06
Frequency of PA/ week	-0.01	0.06	0.04	-0.01	0.06	-0.27
BMI (kg/m2)	0.17	0.33	1.00	0.60	0.76	-0.30
Waist-to-hip ratio	0.04	0.07	0.60	1.00	0.21	-0.02
Fat %	0.25	0.39	0.76	0.21	1.00	-0.51
Muscle %	-0.12	-0.36	-0.30	-0.02	-0.51	1.00
Hs-CRP (mg/l) [2]	0.51	0.46	0.44	0.28	0.48	-0.36
E-DII	0.46	1.00	0.33	0.07	0.39	-0.36

[1] Correlations were significant between (-0.10 to 0.10)

Perceived stress scale (PSS), Saudi riyals (SR), Physical activity (PA), Body mass index (BMI), High sensitivity C reactive protein (hs-CRP), Energy adjusted dietary inflammatory index (E-DII).

[2] Hs-CRP were collected from 289 participants.
